# The rupture risk factors of mirror intracranial aneurysms: A systematic review and meta-analysis based on morphological and hemodynamic parameters

**DOI:** 10.1371/journal.pone.0286249

**Published:** 2023-06-23

**Authors:** Huang Yong-Wei, Xiao-Yi Wang, Zong-Ping Li, Xiao-Shuang Yin

**Affiliations:** 1 Department of Neurosurgery, Mian yang Central Hospital, School of Medicine, University of Electronic Science and Technology of China, Mian yang, Sichuan, People’s Republic of China; 2 Department of Immunology, Mian yang Central Hospital, School of Medicine, University of Electronic Science and Technology of China, Mian yang, Sichuan, People’s Republic of China; Anhui University, CANADA

## Abstract

**Objective:**

Intracranial aneurysms (IAs) are a prevalent form of vascular disease that can lead to fatal outcomes upon rupture. Mirror intracranial aneurysms (MIAs) are a specific type of multiple aneurysms situated symmetrically on both sides of the parent arteries. The factors contributing to the risk of MIA rupture, based on morphological and hemodynamic parameters, are currently controversial. Thus, we conducted a systematic review and meta-analysis to investigate the risk factors for MIA rupture.

**Methods:**

The study performed an electronic search of Chinese and English databases, including China national Knowledge Infrastructure (CNKI), WanFang, VIP, PubMed, Embase, Web of Science, Scopus, and the Cochrane Library databases, and adhered to the Preferred Reporting Items for Systematic Reviews and Meta-Analyses (PRISMA) guidelines. The morphological parameters (IA size, aspect ratio [AR], size ratio [SR], bottleneck factor [BNF], height-width ratio [HWR], irregular shape) and hemodynamic parameters (wall shear stress [WSS], low WSS area [LSA], oscillatory shear index [OSI]) were analyzed for their significance in determining the risk of MIA rupture.

**Results:**

The analysis comprised 18 retrospective studies involving 647 patients, with a total of 1294 IAs detected, including 605 ruptured and 689 unruptured. The meta-analysis revealed that IA size, AR, SR, and irregular shape exhibited significant differences between the ruptured and unruptured groups, but HWR did not. In terms of hemodynamic parameters, WSS, OSI, and LSA were found to have significant differences between the two groups.

**Conclusions:**

Our results demonstrate that larger IAs, higher AR, SR, and BNF are associated with a higher risk of rupture in patients with MIAs, regardless of their location. there is no significant difference in HWR between the ruptured and unruptured groups. These preliminary findings offer valuable insights for clinical decision-making and a more comprehensive comprehension of the current MIA status. Nevertheless, larger and multi-center studies are indispensable for corroborating these findings.

**Systematic review registration:** https://www.crd.york.ac.uk/prospero/ identifier: CRD42022345587.

## Introduction

Intracranial aneurysms (IAs) are a prevalent vascular disorder, and if they rupture, can lead to fatal outcomes. The estimated prevalence of IAs in adults ranges from 2% to 3% **[1]**. Although the rupture rate of IAs is relatively low, between 0.95% to 1.6% **[2, 3]**, sudden rupture can cause severe subarachnoid hemorrhage (SAH), which results in high mortality and severe complications, accounting for 50–60% and 30–40%, respectively **[4]**. Given that surgical interventions, such as coiling and clipping, carry the risk of complications, it is crucial to weigh the risk of rupture against the potential complications when making treatment decisions for unruptured aneurysms **[5]**. Therefore, identifying the rupture risk factors for IAs is crucial to aid in making appropriate treatment decisions.

The exact mechanism behind IA rupture is not fully understood and is considered to be complex. Morphology and hemodynamics have been identified as risk factors for IA pathogenesis, progression, and rupture **[6–8]**. However, previous studies exploring the morphological or hemodynamic risk factors for IA rupture have produced conflicting and controversial results **[9–12]**. For example, Cebral et al. **[11]** found a link between high wall shear stress (WSS) and ruptured IAs, while Xiang et al. **[7]** suggested that low WSS area (LSA) and high oscillatory shear index (OSI) were associated with aneurysm rupture. These differences could be attributed to variations in individual factors and aneurysm locations.

Mirror intracranial aneurysms (MIAs) are a unique type of multiple aneurysms located in the same position on each side of the parent arteries. The rupture of MIAs occurs when one aneurysm ruptures while the other remains intact. MIAs provide an ideal model for studying the parameters associated with rupture. However, the lack of agreement on the rupture-related risk factors for MIAs has limited the ability to make informed treatment decisions. In view of this, we conducted a systematic review and meta-analysis based on morphological and hemodynamic parameters to investigate the rupture risk factors of MIAs. To the best of our knowledge, this is the first meta-analysis to summarize and analyze the rupture risk factors of MIAs.

## Methods

### Aims and PICO statement

This investigation was carried out in adherence to PRISMA (Preferred Reporting Items for Systematic Reviews and Meta-Analyses) guidelines **[13]**. Further information of PRISMA checklist can be found in **S1 Table**. The study protocol was registered with the International Prospective Register of Systematic Reviews (PROSPERO: CRD42022345587) **[14]**. The PICO (Population, Intervention, Comparisons, Outcomes) statement for this study is as follows: Population: Patients diagnosed with Intracranial Aneurysms (MIAs). Intervention: Computational fluid dynamics (CFD) analysis of hemodynamics and assessment of morphology using a 3D-DSA workstation. Comparisons: Comparison between the ruptured and unruptured groups. Outcomes: morphological parameters, which include aneurysm size, aspect ratio (AR), size ratio (SR), bottleneck factor (BNF), height-width ratio (HWR), irregular shape, and hemodynamic parameters (WSS, OSI, LSA). The definitions for these parameters can be found in **S2 Table**.

### Search strategy

In order to identify relevant articles, a systematic search of both Chinese and English databases was conducted. Chinese databases including China national Knowledge Infrastructure (CNKI), Wan Fang, and VIP, as well as English databases such as PubMed, Embase, Web of Science, Scopus, and the Cochrane Library, were searched. Clinical trial registry centers, including clinicaltrials.gov and WHO-ICTRP, were also screened. The search was conducted from the inception of the databases to the end of August 2022 by three reviewers (Huang YW, Yin XS, and Wang XY), using controlled vocabulary (MeSH terms and Emtree) and keywords. The search strategy included the keywords "mirror" AND ("intracranial aneurysm" OR related terms) to ensure all relevant studies were identified. The detailed search strategy is provided in **S3 Table**.

### Inclusion and exclusion criteria

In this study, two independent reviewers (Huang YW and Yin XS) assessed all potential articles to determine their compliance with the inclusion and exclusion criteria. Only peer-reviewed medical journal articles that included patients with MIAs with complete morphological or hemodynamic parameters were considered for inclusion. The comparison of interest was between the ruptured group and the unruptured group, and the outcome measures included morphological parameters (such as the size of the aneurysm, AR, SR, BNF, HWR, and irregular shape) and hemodynamic parameters (WSS, OSI, LSA). Articles such as case reports, reviews, notes, meta-analyses, editorials, letters to the editor, commentaries, and conference abstracts were excluded from this study.

### Data extraction

The extracted data from the included studies were independently reviewed by two reviewers using standardized tables. The following information was extracted: Essential characteristics such as first author, year of publication, country, study design, and number of participants; participant characteristics such as age, gender distribution, location of MIAs, focus of study, modeling of MIAs, hemodynamic parameters, and morphological parameters; and any other relevant data.

### Risk of bias assessment

The potential risk of bias in each study was evaluated using the Newcastle-Ottawa Scale (NOS) tool **[15]**, which assigns a maximum score of nine points. Studies with a score of greater than six points were considered to be of high quality. The evaluation was independently performed by three reviewers (Huang YW, Yin XS, and Li Li ZP), and any disagreements were resolved through group discussions among investigators when necessary.

### Statistical analysis

In order to compare outcomes between ruptured and unruptured groups of MIAs, we conducted a meta-analysis and computed the mean differences (MDs) and 95% confidence intervals (CIs) for continuous variables, and the odds ratios (ORs) and 95% CIs for dichotomous variables. In instances where parameters were reported as median (interquartile), we estimated the mean and standard deviation using sample size, median, range, and interquartile range, following the methods recommended by Luo et al. **[16]** and Wan et al **[17]**. The website https://www.math.hkbu.edu.hk/˜tongt/papers/median2mean.html was used for this purpose. We used the random-effects model of DerSimonian and Laird to account for clinical heterogeneity during meta-analysis **[18]**. We deemed a p-value less than 0.05 to be statistically significant, and assessed the degree of heterogeneity between studies using the Cochrane Q test, with a p-value less than 0.1 or I^2^ greater than 50% indicating significant heterogeneity **[19]**. Statistical analyses were performed using Review Manager software version 5.3.3.

## Result

### Result of literature search and characteristics of eligible studies

**Fig 1:**
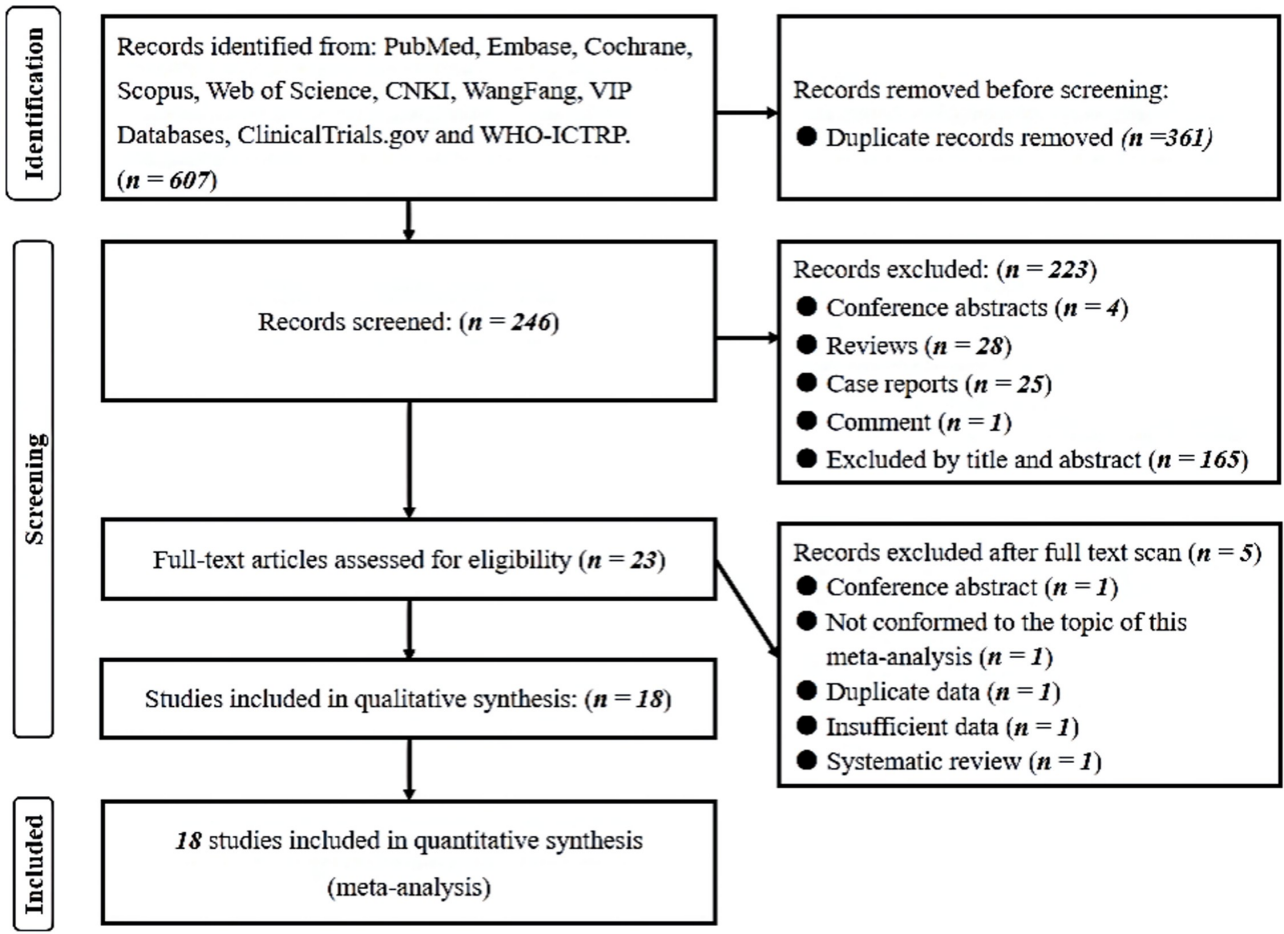
The PRISMA flowchart of included studies.

We conducted an exhaustive search of the literature and identified 607 relevant records. Among these, we subjected 23 articles to full-text evaluation, and ultimately included 18 studies in our systematic review and meta-analysis **[8, 20–36]**. We excluded five articles (one abstract, one on an unrelated topic, one with duplicate data, one with insufficient data, and one systematic review) during the screening process, as shown in the PRISMA flowchart (**Fig 1**). The 18 studies included 647 patients and 1294 IAs, of which 605 were ruptured and 689 were unruptured. The studies comprised 17 single-center and retrospective studies **[8, 20–24, 26, 28, 29, 31–36]** and three multi-center and retrospective studies **[25, 27, 30]**. All studies indicated that the prevalence of MIAs was higher in females than males. We present a summary of our systematic review in **Table 1**.

**Table 1:** The baseline characteristics of included studies.

Author	Year of Publication	Country	Study Design	Participants	Average age (y)	Male (%)	Location of IAs	Study Focus	Modeling of MIAs	Morphological parameters	Hemodynamic parameters
Lu et al	2011	China	retrospective single center **small sample**	9	55.4	44.44	ICA, ACA, MCA	Morphological Hemodynamic	1. Allura FD20 workstation (Philips Healthcare) 2. ICEM CFD 11.0 (ANSYS, Lebanon, New Hampshire)	the IAs size	WSS, LSA, OSI
Li et al	2013	China	retrospective single center	52	56.3	34.62	PComA, MCA, ACA	Morphological	Toshiba Infinix VFi/BP frontal arm system (Toshiba America Medical Systems, Inc., Tustin, CA) 1. In-house software based on the open-source Visualization Tool Kit libraries	D_max width_, D_eck_, H_p_, H_max_, D_v_, AR, SR	—
Xu et al	2013	China	retrospective single center **small sample**	8	63.1	25.00	PComA	Morphological Hemodynamic	1. Allura FD20 workstation (Philips Healthcare) 2. ICEM CFD 11.0 (ANSYS, Lebanon, New Hampshire)	the IAs size, AR, SR, VA, AIA	WSS, LSA, OSI
Fan et al	2015	China	retrospective single center **small sample**	16	60.6	31.25	PComA, MCA, OphA	Morphological Hemodynamic	1. 3D DSA 2. ICEM CFD software (ANSYS Inc., Canonsburg, Pennsylvania, The USA)	the IAs size, D_eck_, D_max width_, AR, BNF, SR, the irregular shape of IAs	WSS, LSA, OSI, Flow stability, Inflow concentration, Impingement size
Jiang et al	2015	China	retrospective single center **small sample**	14	59.1	35.71	PComA	Morphological	3D Slicer	the IAs size, H_max_, D_max width_, D_eck_, AR, BNF, HWR, SR, the irregular shape of IAs	—
Chen et al	2016	China	retrospective multi-center	34	62.2±10.2	14.71	PComA	Morphological	DSA	H_p_, H_max_, D_v_, D_max width_, D_eck_, AR, SR, HWR, AR	
Tian et al	2016	China	retrospective single center	56	58.89	19.64	ICA, MCA	Morphological Hemodynamic	1. 3D DSA 2. ICEM CFD software (ANSYS Inc., Canonsburg, PA, USA)	the IAs size, AR, SR, irregular shape of IAs	WSS, OSI, LSA, flow stability, flow complexity
Huang et al	2017	China	retrospective multi-center	99	62.9±10.2	16.16	PCA, ICA, ACA MCA, PCA	Morphological	CTA	the IAs size, the irregular shape of IAs	—
Doddasomayajula et al	2017	the U. S.	retrospective single center	24	52.9±13.3	12.50	MCA	Morphological Hemodynamic	3D angiography	the IAs size, D_eck_, AR	WSS, LSA, OSI, SCI, V_max_, Q, ICI, VO, SR, VE
Shao et al	2017	China	retrospective single center	22	57.59±10.93	0.00	ICA, MCA, PCA	Morphological	3D DSA	D_max width_, D_eck_, AR, SR	—
Wang et al	2018	China	retrospective multi-center	68	60.68±12.39	14.71	PComA	Morphological	1. CTA (GE LightSpeed VCT; GE Healthcare, Wisconsin, USA) 2. CTA (Toshiba Aquilion One; Toshiba Medical Systems, Tokyo, Japan)	bifurcation, the irregular shape of IAs, the IAs size, H_max_, D_max width_, D_eck_, flow angle, D_v_, AR, HWR, BNF, SR	—
Xu W et al	2020	China	retrospective single center	48	59.1	43.75	MCA	Morphological	N/A	the IAs size, H_max_, D_max width_, D_eck_, D_v_, Wall protrusion AR, HWR, BNF, SR	—
Xu et al	2020	China	retrospective single center	20	54.7±9.66	20.00	MCA	Morphological Hemodynamic	1. CTA 2. ANSYS ICEM 15.0	the IAs size, H_p_, H_max_, D_eck_, AR, SR, BNF, HWR, VA, the irregular shape of IAs, Aneurysm angle, Flow angle, Parent-daughter angle	WSS, LSA, OSI
Yuan et al	2020	China	retrospective single center **small sample**	12	50.67±7.94	41.67	PComA, MCA, OphA	Morphological Hemodynamic	1. 3D DSA (Siemens, Artis Zee Floor VC14). 2. ICEM CFD 14.0 (ANSYS, Canonsburg, PA, US)	the IAs size, AR, SR EI, UI, NSI	WSS, LSA, OSI
Yuan et al	2021	China	retrospective single center	72	58.18±11.22	20.83	PComA	Morphological Hemodynamic	1. 3D-DSA workstation 2. CFD	the IAs size, AR, SR, BNF, HWR, The irregular shape of IAs, Bifurcation, InA, VA, AIA EI, UI, NSI	WSS, LSA, OSI, Flow stability, Inflow concentration Impingement size RRT
Hu et al	2022	China	retrospective single center	29	61.00±11.00	20.69	ICA, MCA, PCA	Morphological Hemodynamic	1. CTA 2. Workbench 15.0 (ANSY Inc., US)	D_max width_, D_eck_, H_max_, D_v_, AR, SR	WSS, LSA, OSI, RRT, AFI
Wang et al	2022	China	retrospective single center	38	54.9±11.3	28.95	MCA	Morphological	CTA	the size of IAs, D_eck_, H_p_, H_max_, AR, SR, Irregular shape of IAs	—
Xin et al	2022	China	retrospective single center	26	56.1±11.0	19.23	MCA	Morphological Hemodynamic	1. CTA 2. ICEM CFD 14 (ANSYS Inc., Canonsburg, PA)	the size of IAs, H_max_ DA, NSI, AR, SR	WSS, LSA, OSI RRT, PLc, EL, sVF

### Meta-analysis and heterogeneity of different endpoints

**Fig 2:**
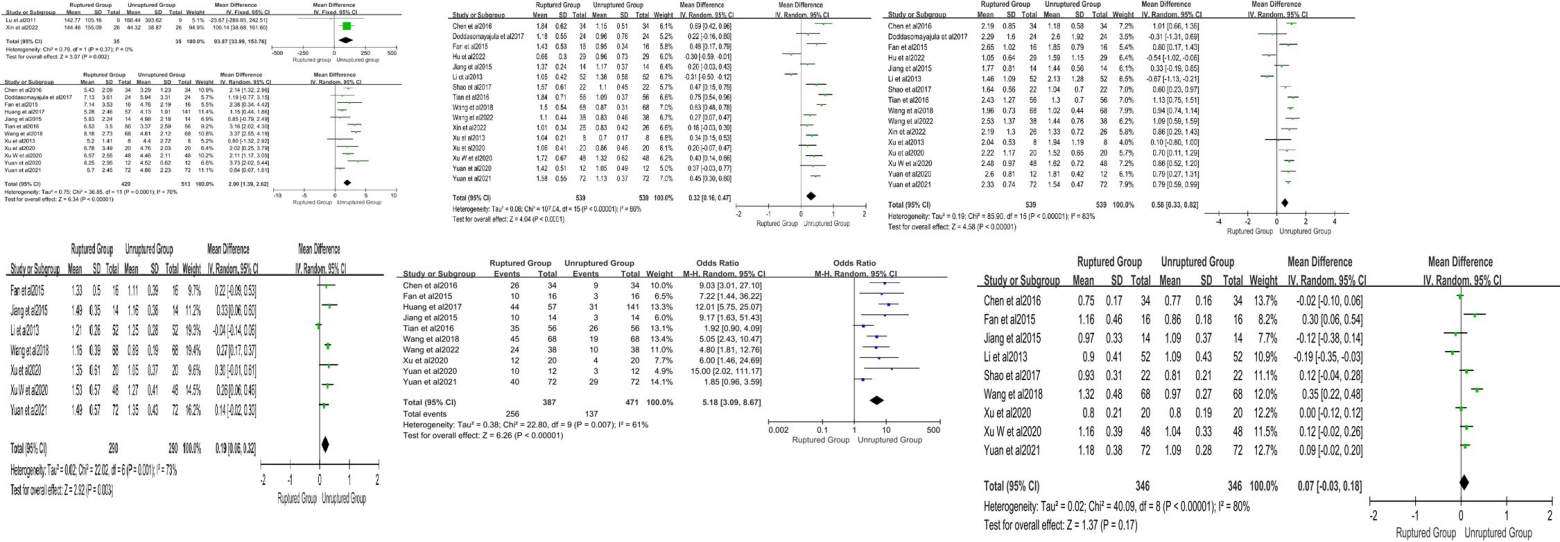
The **(A)** IAs size [mm^3^ and mm], **(B)** AR, **(C)** SR, **(D)** BNF, **(E)** irregular shape, and **(F)** HWR between the ruptured group and unruptured group.

**Fig 3:**
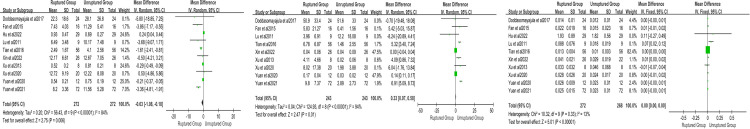
The **(A)** WSS, **(B)** LSA, and **(C)** OSI between ruptured group and unruptured group.

The findings are displayed in **Table 2**, and a meta-analysis was conducted on morphological parameters (**Fig 2A–2F**) and hemodynamic parameters (**Fig 3A–3C**). Our results revealed that several factors, including the size of IAs (mean difference [MD] 2.00; 95% confidence interval [CI] (1.39–2.62); P < 0.00001; I^2^ = 70%), AR (MD 0.32; 95% CI (0.16–0.47); P < 0.0001; I^2^ = 86%), SR (MD 0.58; 95% CI (0.33–0.82); P < 0.00001; I^2^ = 83%), BNF (MD 0.19; 95% CI (0.06–0.32); P = 0.003; I^2^ = 73%), and irregular shape (odds ratio [OR] 5.18; 95% CI (3.09–8.67); P < 0.00001; I^2^ = 61%), were significant risk factors for the rupture of MIAs, except for HWR (MD 0.07; 95% CI (-0.03, 0.18); P = 0.17; I^2^ = 84%). Furthermore, among the hemodynamic parameters, WSS (MD -0.63; 95% CI (-1.08, -0.18); P = 0.006; I^2^ = 84%), OSI (MD 0.00; 95% CI (0.00–0.00); P < 0.00001; I^2^ = 13%), and LSA (MD 0.33; 95% CI (0.07–0.58); P = 0.01; I^2^ = 94%) exhibited significant differences between the ruptured and unruptured groups.

**Table 2:** Meta-analysis and heterogeneity of different endpoints.

Items		Studies, n	Results		
			MD (95% CI)	*P* Value	Heterogeneity (I^2^, *P* for Cochran Q)
Morphology	The IAS volume (mm^3^)	2	93.87 (33.99, 153.76)	P = 0.002	I^2^ = 0%, *P* = 0.37
	The IAS size (mm)	12	2.00 (1.39–2.62)	*P* < 0.00001	I^2^ = 70%, *P* = 0.0001
	AR	16	0.32 (0.16–0.47)	*P* < 0.0001	I^2^ = 86%, *P* < 0.00001
	SR	16	0.58 (0.33–0.82)	*P* < 0.00001	I^2^ = 83%, *P* < 0.00001
	BNF	7	0.19 (0.06–0.32)	*P* = 0.003	I^2^ = 73%, *P* = 0.001
	HER	9	0.07 (-0.03, 0.18)	*P* = 0.17	I^2^ = 84%, *P* < 0.00001
	***Irregular shape***	***10***	***5*.*18 (3*.*09–8*.*67)*** ^***a***^	***P < 0*.*00001***	***I***^***2***^ ***= 61%*, *P = 0*.*007***
Hemodynamics	WSS (Pa)	10	-0.63 (-1.08, -0.18)	*P* = 0.006	I^2^ = 84%, *P* < 0.00001
	LSA	9	0.33 (0.07–0.58)	*P* = 0.01	I^2^ = 94%, *P* < 0.00001
	OSI	10	0.00 (0.00–0.00)	*P* < 0.00001	I^2^ = 13%, *P* = 0.33

**a** Dichotomous variable, OR (95% CI).

In summary, the size of IAs, AR, SR, BNF, irregular shape, WSS, OSI, and LSA were identified as significant risk factors for MIA rupture, with the exception of HWR. However, our results displayed substantial heterogeneity, which persisted even after removing studies individually. The heterogeneity may be due to variations in study design, measurement tools, and other factors.

### Risk of bias assessment

All of the included studies were assessed retrospectively and evaluated using the Newcastle-Ottawa Scale (NOS), with a mean score of 6.78 stars and a standard deviation (SD) of 0.78 stars. The methodological quality of the studies is summarized in **S4 Table**, revealing a low risk of bias in the areas of selection bias, detection bias, and reporting bias. A funnel plot analysis was conducted to assess the possibility of publication bias, and the relevant results are presented in **S1 Fig**.

## Discussion

The rupture risk of intracranial aneurysms (IAs) is determined by both morphological and hemodynamic factors. While many studies have investigated the relationship between these factors and the rupture of microintracranial aneurysms (MIAs), there has been a significant focus on the aneurysm size, which is not a comprehensive approach. To address this limitation, the concept of size ratio (SR) has been introduced as a promising morphological parameter **[37–39]**. SR takes into account both the size of the aneurysm and the diameter of the parent vessel, providing a more complete understanding of the IA’s size and location **[40]**. Several studies have found a significant association between higher SR values and aneurysm rupture in multiple IAs **[41–43]**. However, it should be noted that these studies may have overlooked important variables, such as individual differences and the aneurysm location **[33]**. Therefore, further research that considers these factors is needed to gain a more comprehensive understanding of the relationship between IA morphological and hemodynamic factors and rupture risk.

The study by Ujiie et al. **[44]** defined AR as the ratio of the maximum vertical height (H_p_) to the average neck diameter (D_neck_) of an aneurysm. According to this and other studies, higher AR values were found to be independently associated with an increased risk of aneurysm rupture. As the AR increases, the height of the aneurysm increases or the neck becomes smaller, which can lead to complex flow patterns and slow circulation inside the aneurysm. These changes can trigger inflammation in the aneurysm wall and increase the risk of rupture **[44, 45**]. The study by Li et al. **[21]** found that SR was the sole morphological characteristic linked to IA rupture. They found that H_p_, D_neck_, D_max width_, H_max_, AR, and BNF were significant indicators for ruptured IAs. Jiang et al. **[24]** confirmed that larger size, higher AR, BNF, SR, and irregular shape may contribute to the rupture of PComA aneurysms. Chen et al.’s further study **[25]** discovered that IAs with sizes ≥ 4.5 mm, AR ≥ 1.11, and SR ≥ 1.87 were linked to IA rupture and could be used to evaluate and predict the risk of rupture. However, further studies are needed to confirm the cutoff. In 2017, Huang et al. **[27]** conducted a multi-center study with 99 pairs of MIAs, 57 of which were ruptured and 114 unruptured. However, the study only analyzed patients’ clinical and demographic profiles and limited morphological factors such as IA size and irregular shape. Wang et al. **[30]** conducted a study with 68 patients and found that in PComA MIAs, AR ≥ 0.98 and SR ≥ 1.21 were better predictors of rupture and that anterior dome projection was an independent risk factor for rupture **[31]**.

In terms of hemodynamics, the WSS is crucial to the formation, progression, and rupture of IAs. WSS is a frictional force created by the viscous flow of blood on the vessel surface, and it regulates gene expression and cellular functions in vessel walls via mechanoreceptors in endothelial cells. Low WSS is associated with the degeneration of these cells and can lead to inflammation and an increased risk of rupture. The LSA was found to be another factor related to the rupture risk of IAs, with higher LSA values being associated with a higher risk of rupture. Lu et al.’s study showed that lower WSS, higher LSA, and higher OSI were observed in the ruptured group **[20]**. However, the study had a small sample size, and additional studies are needed to confirm these findings. Other studies have shown that higher LSA values and lower WSS values are associated with an increased risk of IA rupture, both with and without bifurcations **[22, 23]**. Another study found that factors such as lower WSS, higher LSA, and higher AR were all associated with IA rupture and that hemodynamics played a crucial role in determining the rupture status of an aneurysm **[22]**. In 2005, a study exploring both morphological and hemodynamic characteristics showed that higher AR and LSA were reliable indicators for IA rupture. Additionally, SR, HWR, and BNF were found to be higher in ruptured IAs compared to unruptured IAs, with a significant difference between the two groups **[23]**. A study involving 56 pairs of MIAs found a correlation between larger IAs and lower WSS with IA rupture **[26]**. Despite these findings, the author suggests that multi-center and multi-population studies are necessary to strengthen the results. The results were consistent with previous studies that found these two factors to be statistically significant between the ruptured and unruptured groups. Another study found a link between higher OSI and larger ARs with MIA rupture **[28]**. Xu et al.’s study **[32]** focused on MAIs of the MCA and found that larger IAs, higher SR, irregular shape, lower WSS, and higher maximum WSS may contribute to evaluating the risk of MCA IA rupture, regardless of patient clinical features. A study of 72 patients indicated that SR > 1.96 was the most significant parameter associated with IA rupture, while AR, WSS, mean WSS, and LSA all independently indicated IA rupture status **[33]**.

The implications of this study’s findings for clinical practice are substantial. Our results indicate that various morphological and hemodynamic parameters of mirror intracranial aneurysms, such as size, AR, SR, irregular shape, WSS, OSI, and LSA, can be utilized as predictors of rupture risk. This valuable information can assist healthcare providers in making informed decisions regarding the management and treatment of these aneurysms, ultimately leading to improved patient outcomes. By taking into account the specific factors that contribute to rupture risk, medical professionals can develop tailored treatment plans and preventative measures, thus reducing the incidence of life-threatening ruptures. Additionally, our study’s results can guide the development of new screening and monitoring protocols for mirror intracranial aneurysms, facilitating earlier detection and treatment. Overall, these findings have the potential to significantly impact clinical practice and improve patient care.

Our meta-analysis provides a comprehensive and systematic review of existing studies on the morphological and hemodynamic parameters of MIAs, yielding three key findings. Firstly, larger IAs, higher AR, SR, and BNF are associated with a higher risk of rupture in patients with MIAs, regardless of their location. However, we found that the HWR did not significantly differ between the ruptured and unruptured groups. Secondly, most parameters are multi-faceted factors that accurately represent the status of MIAs. Thirdly, the urgent need for further research into hemodynamic factors is highlighted as they have been shown to have severe and devastating consequences. By assessing the morphological and hemodynamic parameters of MIAs, neurosurgeons can identify potential adverse outcomes, such as life-threatening ruptures, earlier. Nonetheless, it is important to acknowledge some limitations of our study, including the predominantly retrospective nature of the analyzed studies, small sample sizes, and a limited geographical representation. Despite these limitations, our findings could be valuable for clinicians in making treatment decisions for patients with MIAs.

### Limitations

It is important to acknowledge the limitations of our meta-analysis. Firstly, the retrospective nature of the majority of the studies analyzed, rather than randomized controlled trials, may affect the strength of our findings. Moreover, some studies had small sample sizes, ranging from 8 to 99 participants. Secondly, the vast majority of the studies (17 out of 18) were conducted by Chinese researchers, highlighting the need for more studies from diverse populations and countries. Finally, the high degree of heterogeneity among the studies analyzed may affect the generalizability of our results. Nevertheless, despite these limitations, we believe that our preliminary findings may still provide clinicians with valuable insights for treating patients with MIAs. Further studies with larger sample sizes and a more diverse population are warranted to validate our results.

## Conclusion

Our results demonstrate that larger IAs, higher AR, SR, and BNF are associated with a higher risk of rupture in patients with MIAs, regardless of their location. there is no significant difference in HWR between the ruptured and unruptured groups. These preliminary findings offer valuable insights for clinical decision-making and a more comprehensive comprehension of the current MIA status. Nevertheless, larger and multi-center studies are indispensable for corroborating these findings.

## Supporting information

S1 Table PRISMA 2020 checklist. (DOC)

S2 Table The meanings of associated parameters. (DOCX)

S3 Table CNKI, WanFang, VIP, PubMed, Embase, Web of Science, Scopus, the Cochrane Library, ClinicalTrials.gov and WHO-ICTRP Search strategy. (DOCX)

S4 Table Quality assessment of included studies by NOS. (DOCX)

S1 Fig Funnel plot of associated parameters. (DOCX)
