# Supplementary material for: The rupture risk factors of mirror intracranial aneurysms: A systematic review and meta-analysis based on morphological and hemodynamic parameters
Source: PLoS One. 2023 Jun 23;18(6):e0286249. doi: 10.1371/journal.pone.0286249 (PMC10289394; doi:10.1371/journal.pone.0286249)
Supplement: S2 Table — (DOCX) [file pone.0286249.s002.docx]

**S2 Table.** The meanings of associated parameters.

| **Parameters** | | **Abbreviation** | **Meanings** |
| --- | --- | --- | --- |
| **morphology** | Hp | — | maximum perpendicular height of IAs |
|  | Hmax | — | the maximum heigh of IAs |
|  | Dmax width | — | maximal width of IAs |
|  | Dneck | — | neck diameter of IAs |
|  | Dv | — | parent artery average diameter |
|  | aspect ratio | **AR** | Hp/Dneck |
|  | size ratio | **SR** | Hmax/D_v_ |
|  | **size** | — | the IAs size |
|  | height width ratio | **HWR** | Hp/Dmax width |
|  | bottleneck factor | **BNF** | Dmax width/Dneck |
|  | vessel angle | VA | — |
|  | aneurysm inclination angle | AIA | — |
|  | inflow angle | InA | — |
|  | ellipticity index | EI | — |
|  | undulation index | UI | — |
|  | non-sphericity index | NSI | — |
|  | **irregular shape** | — | irregular shape of IAs |
| **hemodynamics** | dome area | DA | — |
|  | wall shear stress | **WSS** | — |
|  | low WSS area | **LSA** | — |
|  | oscillatory shear index | **OSI** | — |
|  | relative residence time | **RRT** | — |
|  | aneurysm formation index | **AFI** | — |
|  | pressure loss coefficient | **PLc** | — |
|  | energy loss | **EL** | — |
|  | surface vortex fraction | sVF | — |
|  | shear concentration index | SCI | — |
|  | maximum flow velocity | V_max_ | — |
|  | aneurysm flow rate | Q | — |
|  | inflow concentration index | ICI | — |
|  | mean aneurysm vorticity | VO | — |
|  | shear rates | SR | — |
|  | Mean aneurysm velocity | VE | — |
