# Supplementary material for: The rupture risk factors of mirror intracranial aneurysms: A systematic review and meta-analysis based on morphological and hemodynamic parameters
Source: PLoS One. 2023 Jun 23;18(6):e0286249. doi: 10.1371/journal.pone.0286249 (PMC10289394; doi:10.1371/journal.pone.0286249)
Supplement: S3 Table — (DOCX) [file pone.0286249.s003.docx]

[S3 Table.](#_Toc49954750) CNKI, WanFang, VIP, PubMed, Embase, Web of Science, Scopus, the Cochrane Library, ClinicalTrials.gov and WHO-ICTRP Search strategy.

| **Database** | **Search Strategy** | **Records** |
| --- | --- | --- |
| CNKI | “镜像动脉瘤”[主题] | 40 |
| WanFang | “镜像动脉瘤”[主题] | 20 |
| VIP | “镜像动脉瘤”[主题] | 20 |
| PubMed | “Mirror [Abstract/Title]”AND (intracranial aneurysm [MeSH term] OR its free words [Abstract/Title]) | 120 |
| Embase | ‘mirror’: ab,ti AND (intracranial aneurysm/exp OR ‘its free words’:ab,ti) | 122 |
| Web of Science | mirror[topic] AND intracranial[topic] AND aneurysm[topic] | 159 |
| Scopus | ‘mirror’: ab,ti,kw AND ‘intracranial aneurysm’:ab,ti,kw | 126 |
| Cochrane Library | (mirror): ab,ti,kw AND (intracranial aneurysm[MeSH term] OR ‘its free words’:ab,ti,kw) | 0 |
| Clinicaltrials.gov | mirror AND intracranial aneurysm | 0 |
| WHO-ICTRP | mirror AND intracranial aneurysm | 0 |
| Total | | 607 |
