# Supplementary material for: The rupture risk factors of mirror intracranial aneurysms: A systematic review and meta-analysis based on morphological and hemodynamic parameters
Source: PLoS One. 2023 Jun 23;18(6):e0286249. doi: 10.1371/journal.pone.0286249 (PMC10289394; doi:10.1371/journal.pone.0286249)
Supplement: S4 Table — (DOCX) [file pone.0286249.s004.docx]

# [S4 Table. Quality assessment of included studies by NOS](#_Toc49954757).

| Study ID | Year | Selection | | | | Comparability | | Exposure | | | Stars |
| --- | --- | --- | --- | --- | --- | --- | --- | --- | --- | --- | --- |
|  |  | Q1 | Q2 | Q3 | Q4 | Q5 | Q6 | Q7 | Q8 | Q9 |  |
| Lu et al | 2011 | - | * | * | - | * | * | * | * | * | 7 |
| Li et al | 2013 | - | * | * | - | * | * | * | * | * | 7 |
| Xu et al | 2013 | - | * | * | - | * | * | * | * | * | 7 |
| Fan et al | 2015 | - | * | * | - | * | - | * | * | * | 6 |
| Jiang et al | 2015 | - | * | * | - | * | - | * | * | * | 6 |
| Chen et al | 2016 | - | * | * | - | * | * | * | * | * | 7 |
| Tian et al | 2016 | - | * | * | - | * | * | * | * | * | 7 |
| Huang et al | 2017 | * | * | * | - | * | * | * | * | * | 8 |
| Doddasomayajula et al | 2017 | - | * | * | - | * | * | * | * | * | 7 |
| Shao et al | 2017 | - | * | * | - | * | - | * | * | * | 6 |
| Wang et al | 2018 | * | * | * | - | * | * | * | * | * | 8 |
| Xu W et al | 2020 | * | * | * | - | * | * | * | * | * | 8 |
| Xu et al | 2020 | - | * | * | - | * | - | * | * | * | 6 |
| Yuan et al | 2020 | - | * | * | - | * | * | * | * | * | 7 |
| Yuan et al | 2021 | - | * | * | - | * | * | * | * | * | 7 |
| Hu et al | 2022 | - | * | * | - | * | - | * | * | * | 6 |
| Wang et al | 2022 | - | * | * | - | * | - | * | * | * | 6 |
| Xin et al | 2022 | - | * | * | - | * | - | * | * | * | 6 |

**Q1** Is the case definition adequate?

**Q2** Representativeness of the cases.

**Q3** Selection of Controls.

**Q4** Definition of Controls.

**Q5** study controls for the most important factor.

**Q6** study controls for any additional factor.

**Q7** Ascertainment of exposure.

**Q8** Same method of ascertainment for cases and controls.

**Q9** Non-Response rate.
