# Supplementary material for: The rupture risk factors of mirror intracranial aneurysms: A systematic review and meta-analysis based on morphological and hemodynamic parameters
Source: PLoS One. 2023 Jun 23;18(6):e0286249. doi: 10.1371/journal.pone.0286249 (PMC10289394; doi:10.1371/journal.pone.0286249)

**S1 Fig.** Funnel plot of associated parameters.

1. The IAs size [mm^3^ and mm]


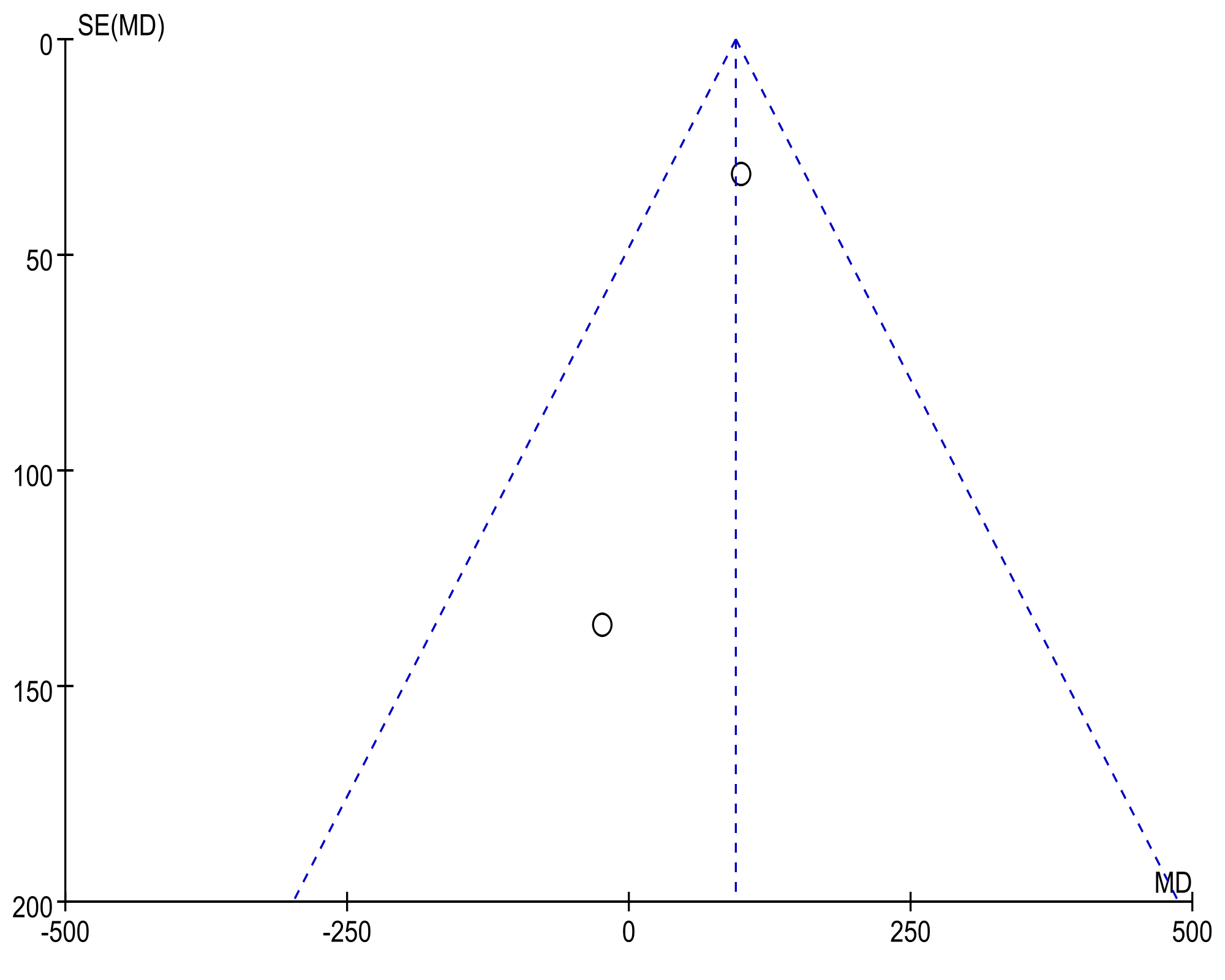

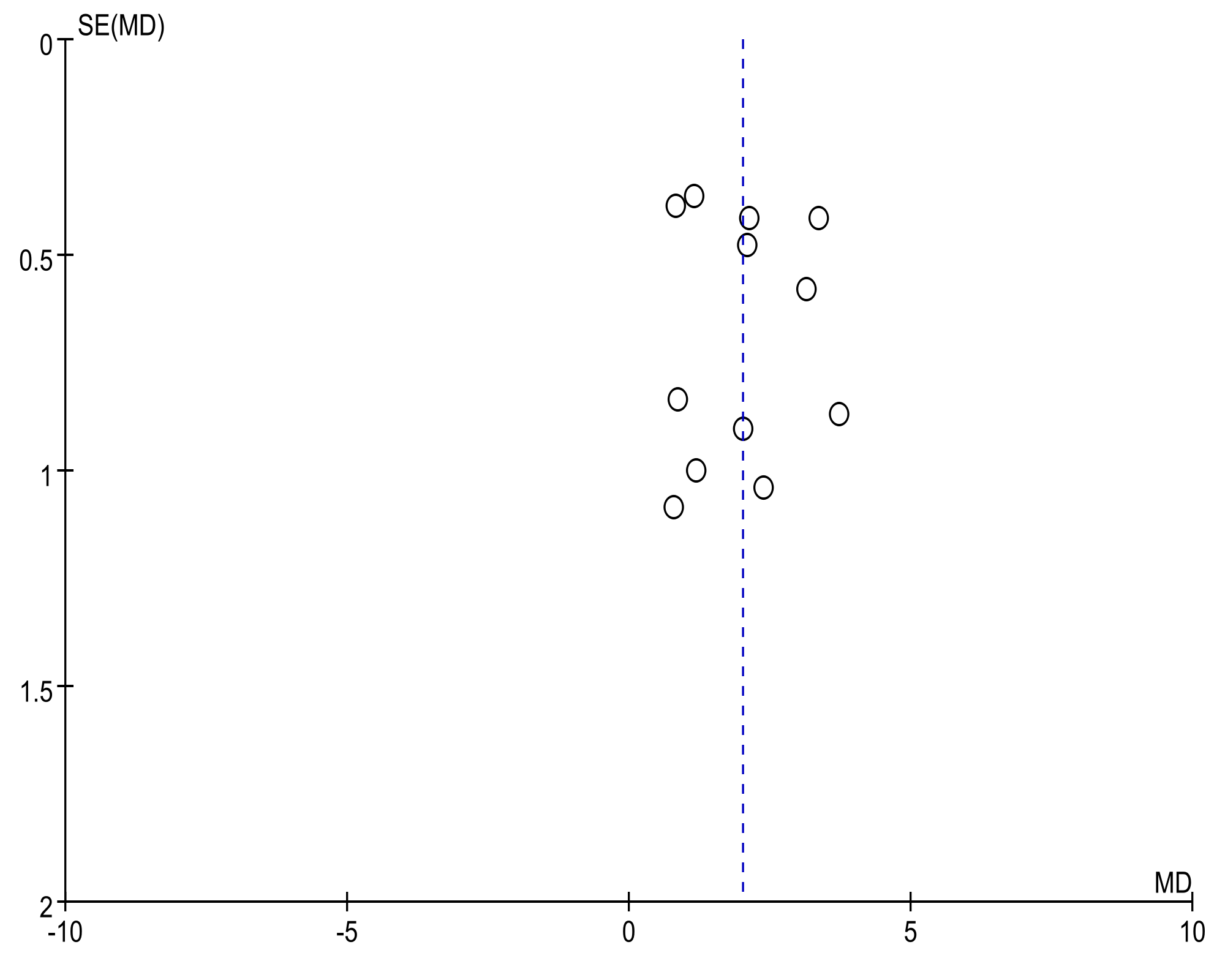


1. AR


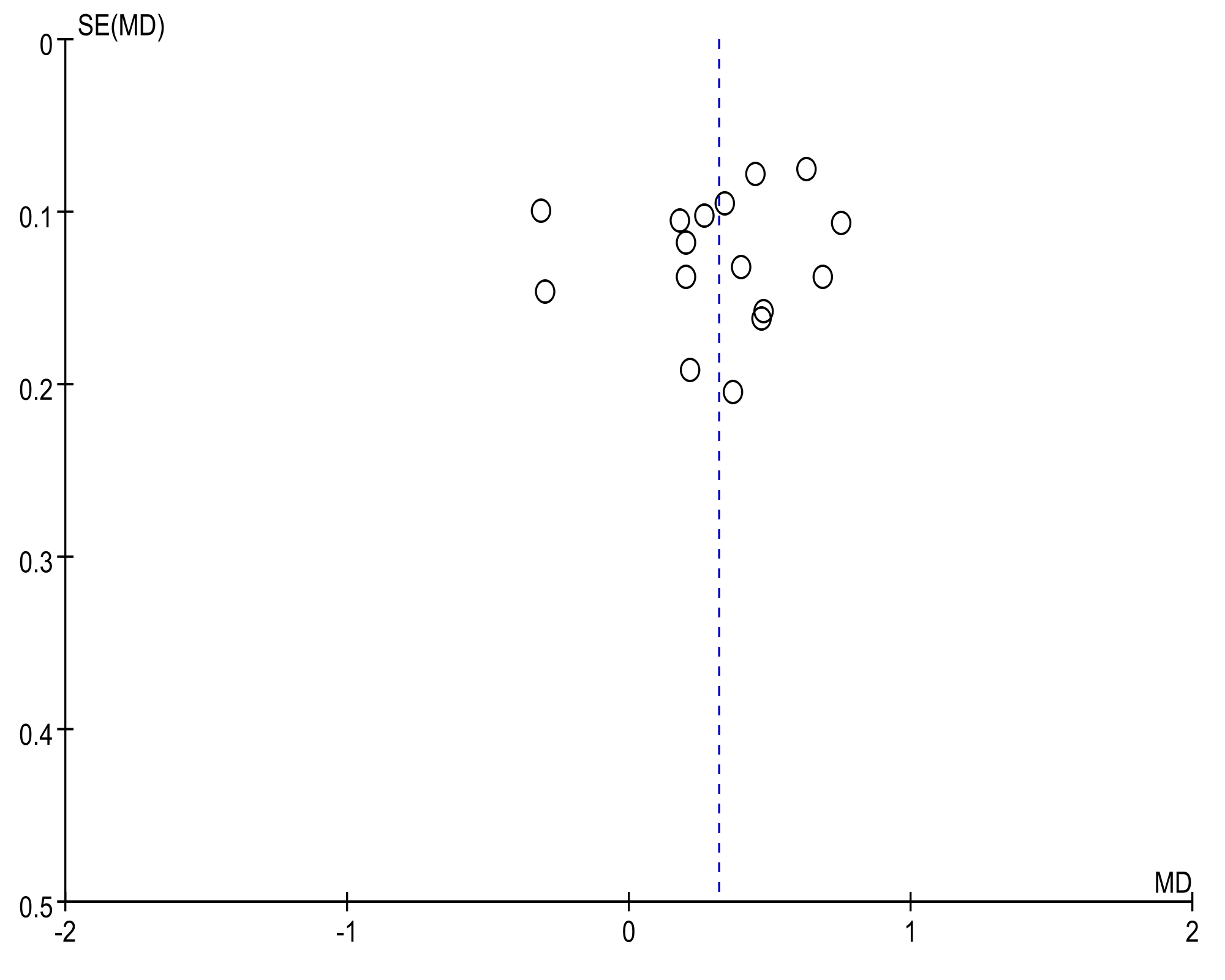


1. SR


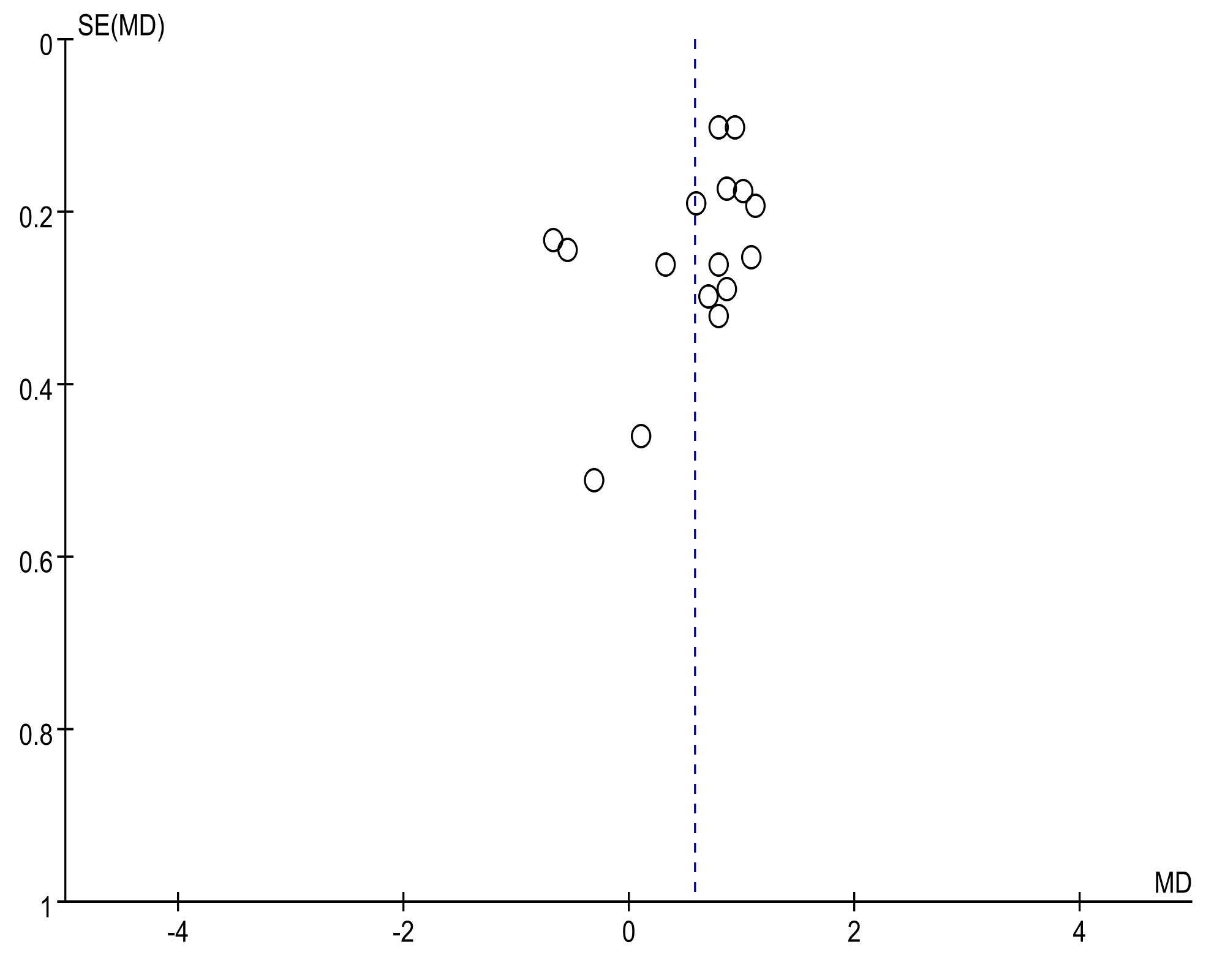


1. BNF


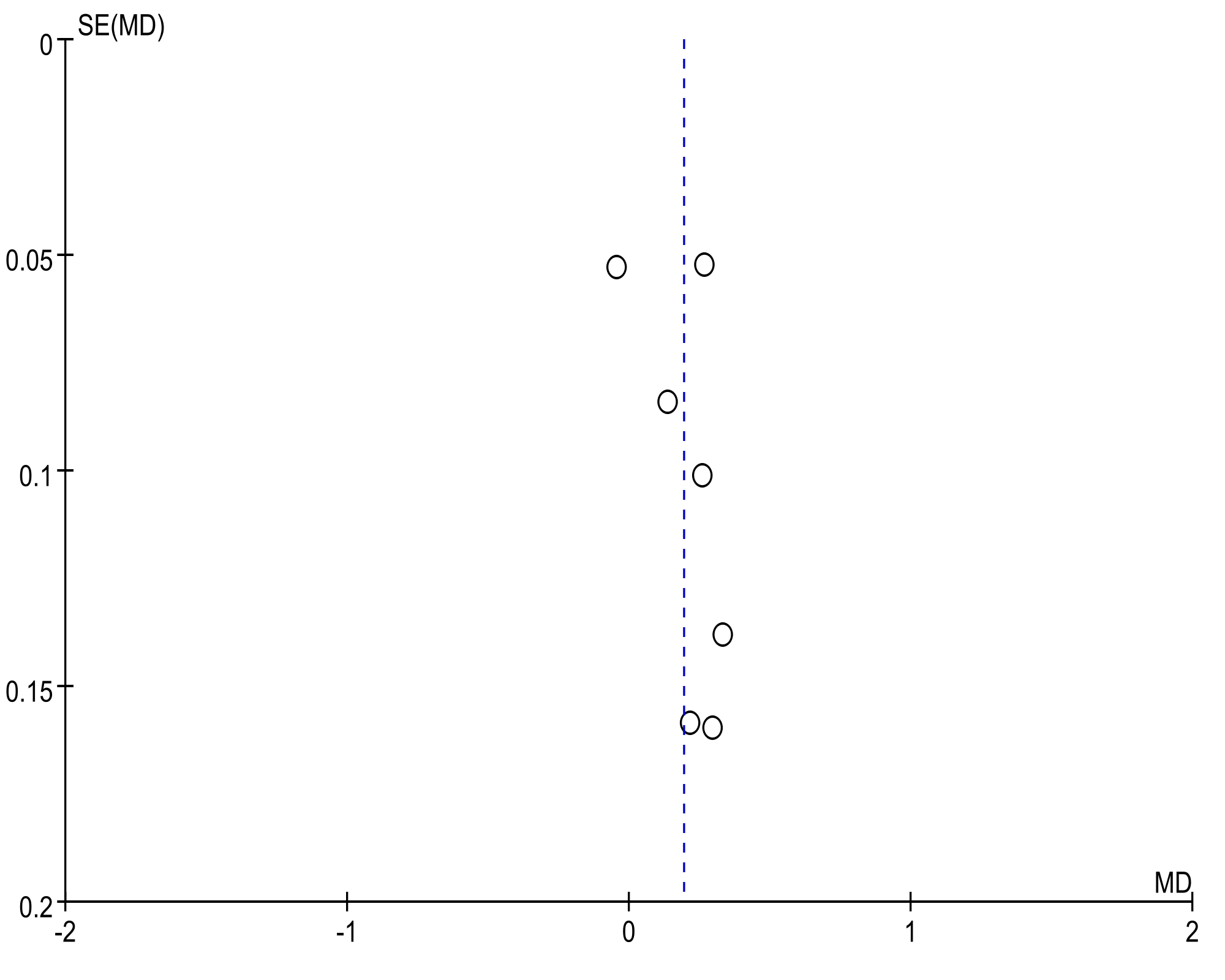


1. irregular shape


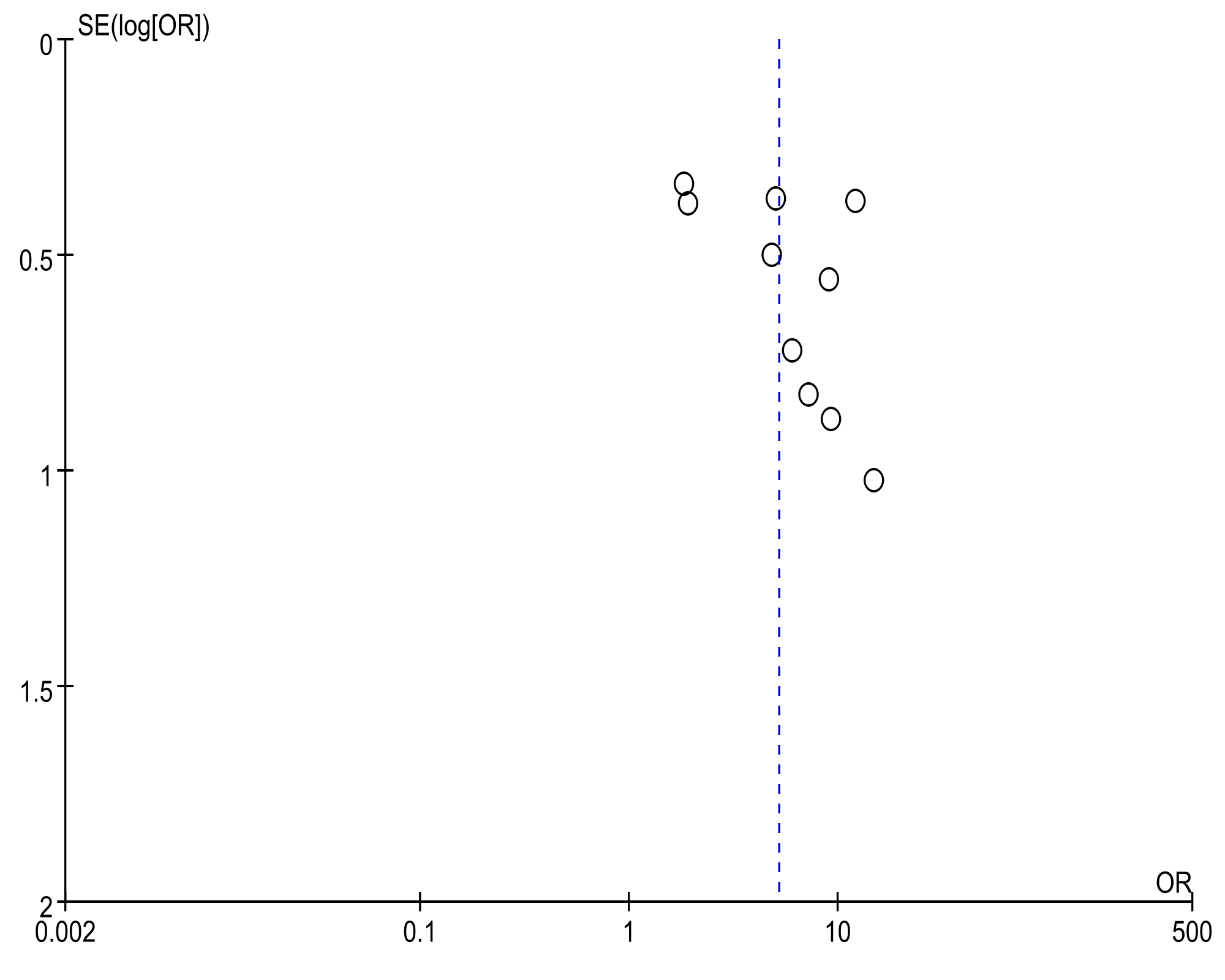


1. HWR


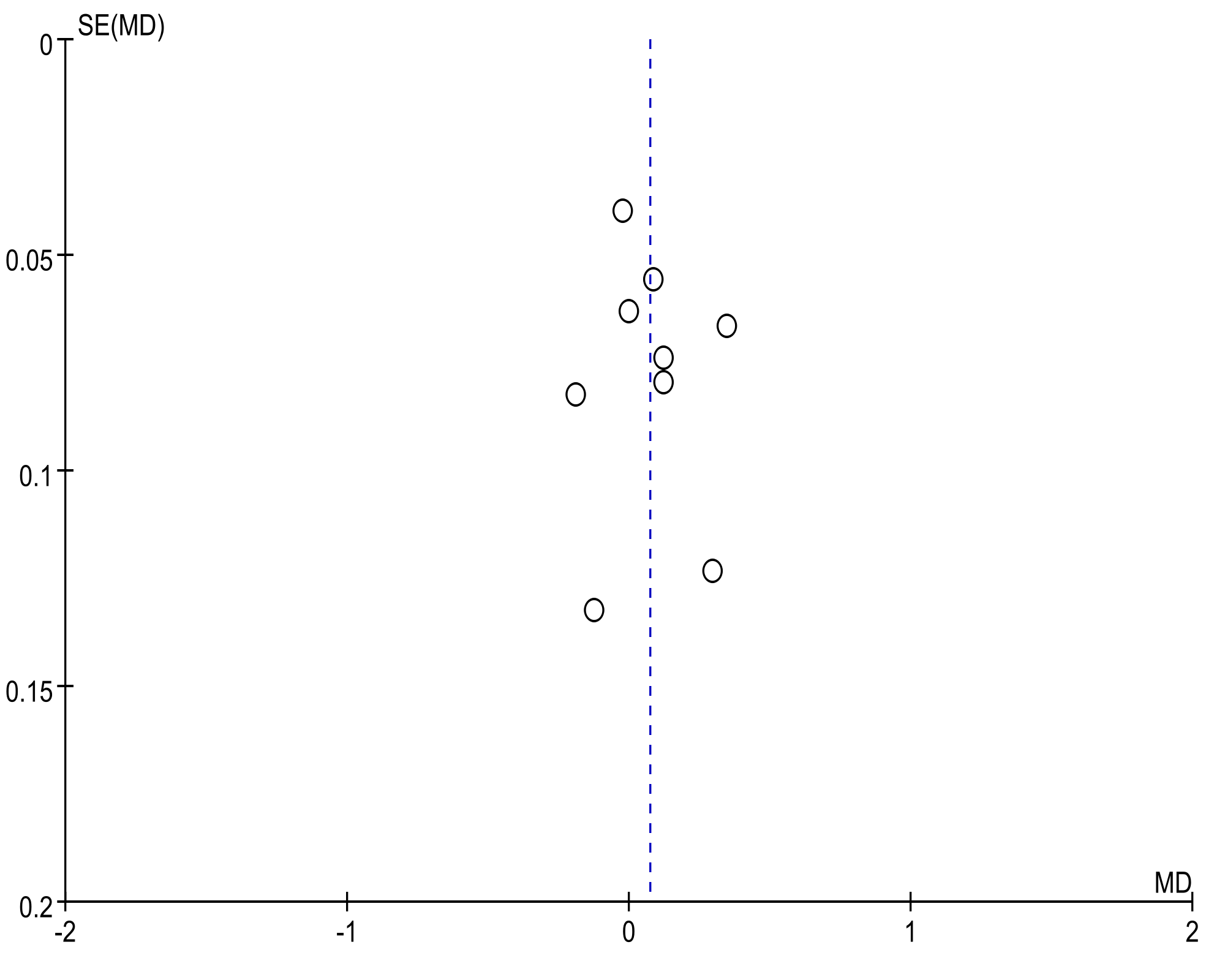


1. WSS


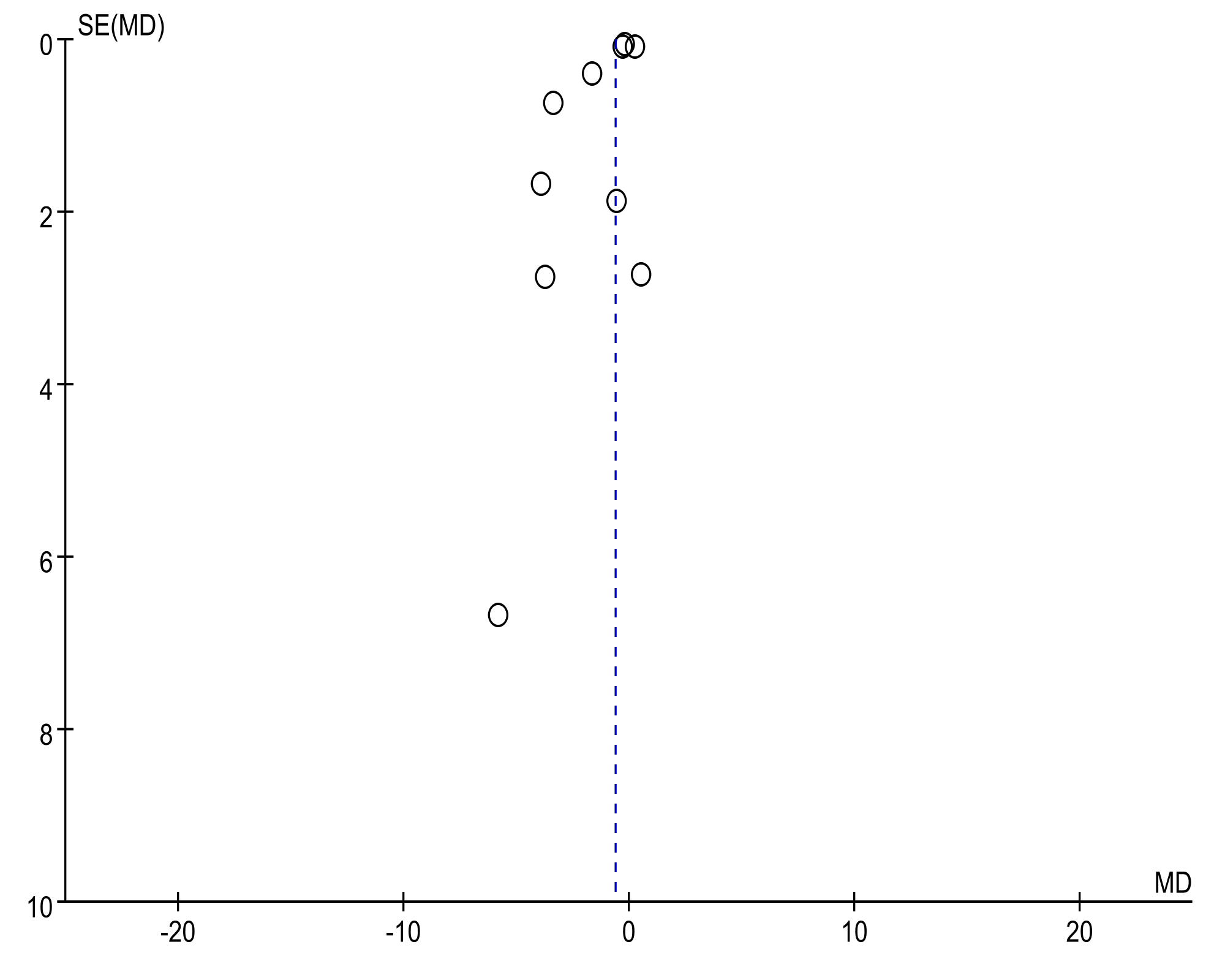


1. LSA


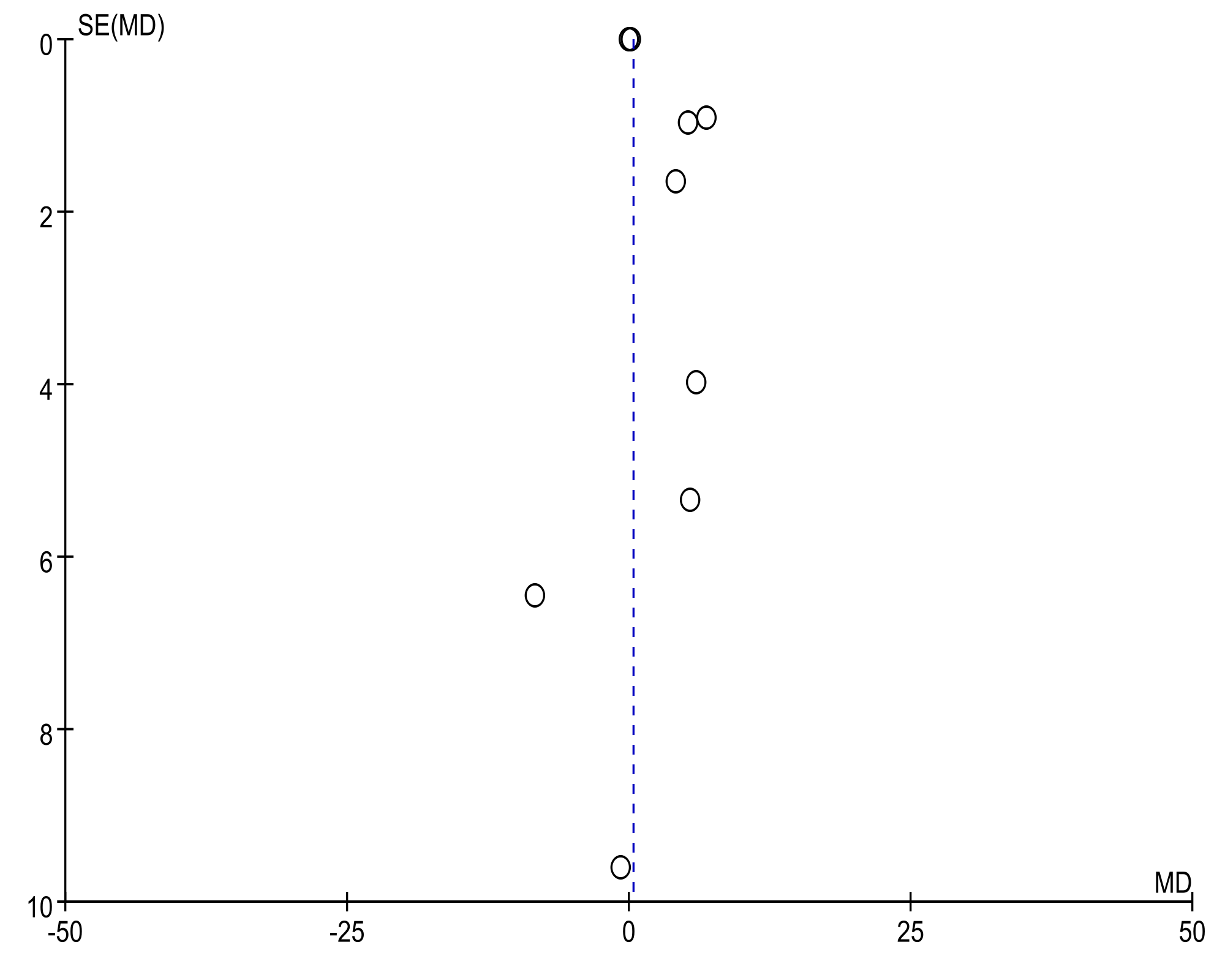


1. OSI


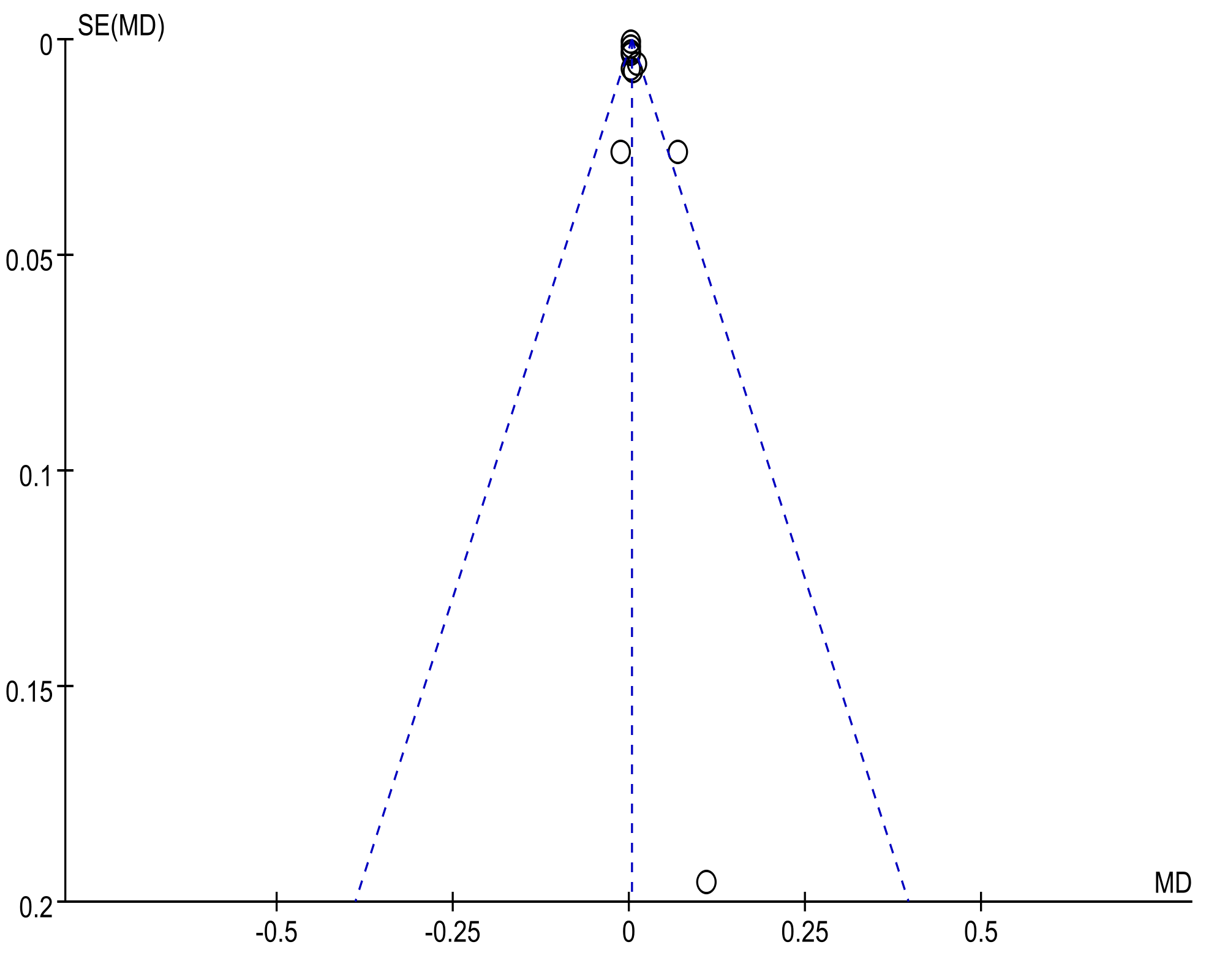

Supplement: S1 Fig — (DOCX) [file pone.0286249.s005.docx]
